# Prevalence and Morphology of the Coracoclavicular Joint: An Osteological Study of 2,724 Subjects Using Univariable and Multivariable Logistic Regression Analyses

**DOI:** 10.3389/fsurg.2021.761441

**Published:** 2021-10-28

**Authors:** Ethan Robert Harlow, Lee M. Sasala, Christopher E. Talbot, Bijal J. Desai, Jason Ina, Shana Miskovsky

**Affiliations:** ^1^Department of Orthopaedic Surgery, University Hospitals Cleveland Medical Center, Cleveland, OH, United States; ^2^Case Western Reserve School of Medicine, Cleveland, OH, United States; ^3^Department of Neurological Surgery, Rutgers New Jersey Medical School, Newark, NJ, United States; ^4^Sports Medicine Institute, University Hospitals Cleveland Medical Center, Cleveland, OH, United States

**Keywords:** coracoclavicular joint, anterior shoulder pain, osteological abnormalities, clavicle anatomy, coracoid anatomy, population variation model, shoulder impingement

## Abstract

**Background:** The coracoclavicular joint (CCJ) is an anomalous articulation between the surfaces of the inferior clavicle and superior coracoid and its etiology is controversial. Reportedly, symptomatic patients demonstrate significant functional limitations including shoulder abduction loss and potential for brachial plexus compression and impingement.

**Purpose:** To determine the prevalence of CCJ across age, gender and ethnicity, and to identify clinically useful morphological characteristics.

**Methods:** 2,724 subjects with intact clavicles and scapulae from the Hamann-Todd Osteological Collection were evaluated for the presence of CCJ. Logistic regression was used to determine the effect of age, height, gender, and race on prevalence of CCJ. 354 clavicles with CCJ were measured for size and location of the CCJ facet.

**Results:** CCJ was observed in 9% of subjects. CCJ was more prevalent in African-Americans (12%) than Caucasian-Americans (6%) (*p* < 0.001) and more prevalent in females (11%) than males (8%) (*p* = 0.055). Facet location along clavicle length was consistent (average 25%, range 15–35%). But, facet location along clavicle width varied (average 60%, range 10–90%), with males having a more posterior location. For every 10-year increase in age, facet elevation (*p* = 0.001) and surface area (*p* < 0.001) increased.

**Conclusions:** CCJ prevalence was 9% in our large osseous population, found more commonly in African-Americans and females. Facet location is predictable with respect to clavicle length, but less so along clavicle width. The clavicular facet may develop at some point in life and continue to grow in size after its appearance.

**Clinical Relevance:** Presence of a CCJ represents a potential overlooked source of anterior shoulder pain and supracoracoid impingement. Epidemiologic and morphological characteristics presented in our study can aid in the identification, clinical understanding, and surgical excision of a symptomatic CCJ. Level of Evidence: Level IV.

## Introduction

The coracoclavicular joint (CCJ) is an anomalous articulation between the horizontal part of the coracoid process of the scapula and the inferior surface of the clavicle. This articulation was first observed in the 19th century through cadaveric dissection and osteological studies have labeled the presence of a round and bulging osseous clavicular facet on the conoid tubercle distinct from the cone-shaped ossification of the conoid ligament usually with an associated osseous facet on the superior surface of the horizontal part of the coracoid process as a CCJ (1, 2). Subsequently, a few cadaveric, osteologic, and radiographical studies have reported frequencies of CCJ ranging widely from 0.04–40.7% (3–7). The definition and etiology of the CCJ has been debated in the literature. In wet dissection, the presence of cartilaginous facets on the clavicle and coracoid indicative of a diarthrotic synovial joint have been found and identified as a CCJ; moreover, the presence of a bony facet on the clavicle articulating with the coracoid without well-defined cartilage or capsule has also been classified as a CCJ (8, 9). Interestingly, authors have argued the CCJ is a congenital anomaly with autosomal dominant inheritance while others have endorsed occupational stresses and degeneration associated with the aging process as contributing factors to its development (9–11). It is evident that the presence of bony articulating facets on the clavicle and coracoid are essential in defining a CCJ, yet it is unclear and inconsistent across studies on whether or not it represents a true joint or is more related to osseous sequelae from mechanical causes.

A CCJ as a source of anterior shoulder pain may be encountered clinically more often than previously appreciated and some authors have proposed that symptomatic cases are grossly underreported (12). This could be due to a lack of awareness of its presence among orthopedic surgeons, as most textbooks do not describe the CCJ (12). However, knowledge of this anatomical finding is crucial in the management of anterior shoulder pain without an obvious alternative cause. There have been 17 cases of symptomatic CCJ described in the literature (9, 12–27). These patients sustained a precipitating trauma to the involved shoulder and presented with recurrent anterior shoulder pain (3, 19). Symptomatic patients also demonstrated significant functional limitations including loss of shoulder abduction and flexion, brachial plexus compression, supracoracoid impingement and thoracic outlet syndrome (6, 12, 25). In most instances, surgical excision of the CCJ was required and allowed for complete resolution of symptoms (12).

Previous osteological studies have compared the morphometric variables of the clavicle and scapula in individuals with and without a CCJ. One study found that individuals with CCJ have larger scapulae, longer clavicles, and longer first ribs (5). A later study found no morphometric differences between individuals with and without a CCJ (10). However, no previous studies have specifically sought to characterize the morphology of the CCJ.

Due to the limited and contradictory data currently available in the literature, our study's purpose is to (1) estimate the prevalence and population trends of CCJ based on a large sample of osseous specimens, to (2) describe morphological characteristics of the CCJ, and to (3) determine if there is a relationship between CCJ prevalence and increasing age.

## Materials and Methods

### Specimens

**Figure 1:**
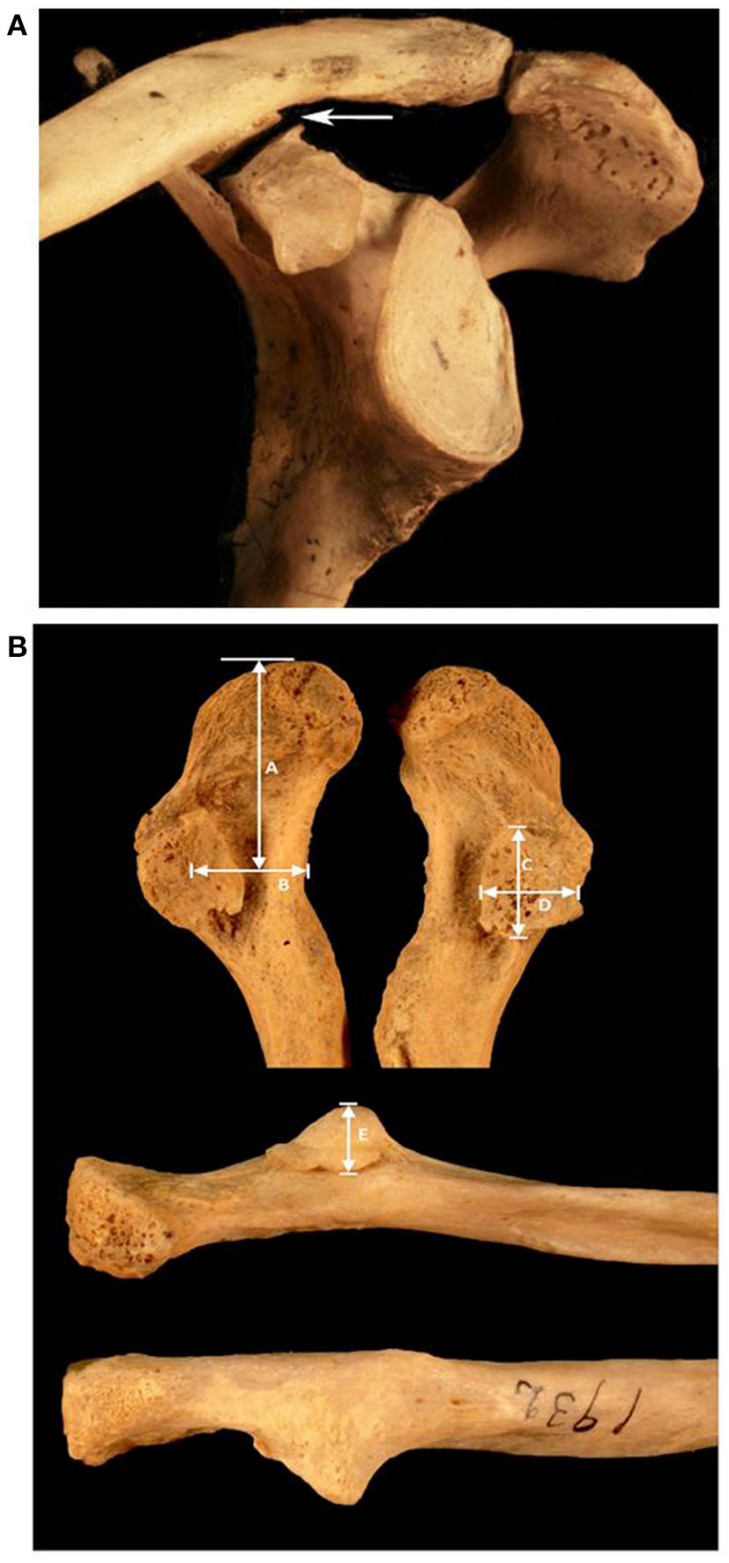
**(A)** Articulated CCJ. This image is an anterolateral view of an articulated scapula and clavicle, with an arrow pointing to the CCJ. **(B)** Clavicular facet. The upper image shows an inferior view of the clavicles from an individual with bilateral facets. The lower images show the elevation of the facet in anterior (middle image) and posterior views. Morphologic measurements include A: distance from acromio-clavicular joint to midpoint of facet, B: distance from anterior border of clavicle to midpoint of facet, C: facet length, D: facet width, E: facet elevation.

Clavicles and scapulae were obtained from the Hamann-Todd Osteological Collection at the Cleveland Museum of Natural History (Cleveland, OH). This collection consists of complete, cleaned human skeletons of unclaimed status from the Cleveland city morgue between 1912 and 1938. Use of these specimens does not require institutional review board approval as it is a publicly available collection. All specimens with a known age and race were included. 245 specimens had to be excluded due to significant specimen damage, a missing clavicle or scapula, evidence of fracture, or extensive arthritic changes to the clavicle precluding measurements. A total of 2,724 subjects (2,252 males, 472 females) were included in this study. One specimen with bilateral CCJ facets had a damaged clavicle that did not allow for measurement. This clavicle was included in the prevalence analysis, but was excluded from the morphological analysis. The Hamann-Todd Collection provided clinical data at time of death for the samples including age, gender, height, and race (Table 1). A representative example of a CCJ is shown in Figures 1A,B.

**Table 1:** Subject demographics.

**Variable**	**Summary statistic**
Age	47.9 ± 16.2 Range = (1, 105)
Age>30	2,309 (84.8)
Gender=male	2,252 (82.7)
Race=AA	1,098 (40.3)
Height (mm)	1,696 ± 116 Range = (645, 1,985)
CC Facet	233 (8.6)
Bilateral CCJ	122 (52.4)
Unilateral Right	60 (25.8)
Unilateral Left	51 (21.9)

*Continuous variables are reported as mean ± SD (and range), and categorical variables are reported as frequency (%) for all subjects (n = 2,724). Frequency of bilateral or unilateral CCJ is reported as frequency (%) for subjects with a CCJ*.

### Measurements

Two authors (LS and CT) performed the assessments and measurements. The clavicles of all specimens were examined for the presence of a CCJ facet. This was defined as a round and bulging structure on the conoid tubercle of the clavicle different from the smaller cone-shaped ossified structure representing conoid ligament using methodology similar to that of Gumina et al. (2). For specimens with a CCJ facet, measurements of the clavicle were taken using digital calipers (Mitutoyo, Kangawa, Japan) with 0.01 mm accuracy. Clavicle length, too large to be measured by calipers, was measured as the straight-line distance between sternal and acromial ends using an osteometric board fitted with digital calipers (Mitutoyo, Kangawa, Japan) with the same accuracy.

The distance was measured from the acromioclavicular (AC) joint facet to the center of the observed clavicular facet (CC facet) along the long axis of the clavicle. The clavicular width at the center location of the CC facet was measured. Along the same anterior-posterior axis of the clavicular width, the distance was measured from the anterior margin of the clavicle to the center of the facet. Facet length (*fL*) and breadth (*fB*) were measured on the longest and shortest axes to approximate facet surface area by treating the facet as an ellipse where area is equal to (*fL*/2)(*fB*/2)π. A third axis was measured to describe the elevation or height of the facet from the normal surface of the clavicle (Figure 1B).

### Statistical Analysis

Variables measured at the subject level were summarized with means and standard deviations for continuous variables, and with frequencies and percentages for categorical variables. Prevalence of the CCJ (present in either clavicle for each subject) was modeled using univariate and multivariable logistic regression to determine the effect of age, height, gender, and race. The best multivariable model was chosen from among all possible subsets including all two-way interactions based on the model that had the lowest Schwarz's Bayesian Information Criterion (BIC). Age was treated as a dichotomous variable with a cutoff of 30 years; an age at which a person's joints would be theoretically fully developed unless influenced by environmental effects. The same analysis strategy was used for modeling the prevalence of bilateral CCJ.

Size and location of CC facets were measured at the clavicle level on those clavicles for which there was a facet present. Size was measured by elevation and surface area, which were normalized using clavicle length. Location was measured by the midpoint of the facet along clavicle length and the midpoint of the facet along clavicle width, which were normalized using clavicle length and width, respectively. Results obtained were then multiplied by 100 to increase readability and interpretability after the normalization process. The effects of age, gender, and race on morphologic variables were estimated with regression models utilizing generalized estimating equations (GEE) with an exchangeable correlation structure to account for the expected correlation within subjects who had more than one facet. In this analysis, age was treated as a continuous variable because the size of the facet would be expected to increase over time (if age is an important predictor of size). All statistical analyses were performed using R (R Foundation for Statistical Computing, Vienna, Austria).

## Results

A CCJ was observed in 233 (8.6%) of individuals examined. Of the individuals having a CCJ, 122 (52.4%) expressed bilaterally, 60 (25.8%) were right unilateral, and 51 (21.9%) were left unilateral (Table 1). The prevalence of a CCJ was 8.1% in males and 10.8% in females (Table 2). There was a prevalence of 11.7% in African-Americans and 6.4% in Caucasians: this was the only statistically significant difference in the prevalence of CCJ among demographic groups [OR = 1.95 (1.49, 2.55), *p* < 0.001]. A bilateral CCJ was present in 4.5% of individuals examined. Males had a statistically significantly lower prevalence of bilateral CCJ (4.1%) than females (6.4%) [OR = 0.63 (0.41, 0.96), *p* = 0.031]. African-Americans (6.6%) had a significantly higher prevalence of bilateral CCJ than Caucasians (3.0%) [OR = 2.29 (1.58, 3.32), *p* < 0,001]. There was no significant difference in prevalence of CCJ according to gender in the multivariable model.

**Table 2:** Prevalence of Coracoclavicular Joint (CCJ).

**Effect**	**Prevalence in Reference group**	**Prevalence in Comparative group**	**Effect size OR (CI)**	***p*-value**
Total CCJ – Unilateral or Bilateral				
Overall	233/2,724 = 8.6%		–	–
10 year increase in age	–	–	0.98 (0.91, 1.07)	0.696
Age greater than 30	26/415 = 6.3%	207/2,309 = 9.0%	1.47 (0.97, 2.25)	0.072
Male gender	51/472 = 10.8%	182/2,252 = 8.1%	0.73 (0.52, 1.01)	0.055
AA race	104/1,626 = 6.4%	129/1,098 = 11.7%	1.95 (1.49, 2.55)	<0.001
100mm increase in height	–	–	1.12 (0.98, 1.28)	0.093
Bilateral CCJ				
Overall	122/2,724 = 4.5%		–	–
10 year increase in age	–	–	1.00 (0.89, 1.11)	0.942
Age greater than 30	12/415 = 2.9%	110/2,309 = 4.8%	1.68 (0.92, 3.08)	0.093
Male gender	30/472 = 6.4%	92/2,252 = 4.1%	0.63 (0.41, 0.96)	0.031
AA race	49/1,626 = 3.0%	73/1,098 = 6.6%	2.29 (1.58, 3.32)	<0.001
100mm increase in height	–	–	1.18 (0.97, 1.43)	0.089
Multivariate modeling of CCJ				
Total CCJ – Unilateral or Bilateral				
AA and age>30	125/1921 = 6.5%	108/803 = 13.4%	2.23 (1.70, 2.93)	<0.001
Bilateral CCJ				
AA and age>30	59/1921 = 3.1%	63/803 = 7.8%	2.69 (1.86, 3.87)	<0.001

*This table displays the results of 3 different analyses for prevalence of the CCJ, total CCJ, bilateral CCJ, and multivariate modeling. For each dichotomous variable, prevalence is reported as both a total number and percentage. The reference group is the group for which the effect is false (e.g. for male gender effect, the reference group is female) and the comparative group is the group for which the effect is true (e.g. for male gender effect, the comparative group is male). Age was treated as both a continuous variable and a dichotomous variable with a cutoff of 30 years. The multivariate model considered 2-way interactions. Odds ratios(OR) of having a CCJ in the comparative group relative to the reference group are reported along with 95% confidence intervals and p-values*.

Table 3 shows the summary measures for subject demographics and morphological characteristics and measurements for all observable facets. The normalized CC facet midpoint along clavicle width (anterior to posterior) using the anterior border of the clavicle as the reference point (Table 4) was significantly different between males (60.4 ± 14.8) and females (54.4 ± 16.8, *p* = 0.017), but not with respect to age or race. CC facet position along the width of the clavicle varied in all groups (range 10–90%), but was located consistently more posteriorly in males. With regard to CC facet midpoint along clavicle length (lateral to medial) using the AC joint as the reference point, there were no significant differences according to age, race or gender. However, its location was more consistent, approximately 25% along the length on average from the AC joint (range 15–35%) (**Figure 3**).

**Table 3:** Morphology summary measures.

**Demographic**	**Summary measure (*n* = 354)**	
Age	47.5 ± 14.4 Range = (18, 86)	
Gender = male	274 (77.4)	
Race = AA	203 (57.3)	
Measurement	Raw (mm/ mm^2^)	Normalized by L (or W)
Clavicle length	151.0 ± 11.7	–
Clavicle width	18.2 ± 3.2	–
CC facet elevation	1.8 ± 1.3	1.2 ± 0.9
CC facet surface area	79.2 ± 41.5	52.3 ± 27.0
CC facet midpoint length	37.9 ± 4.8	25.1 ± 2.9
CC facet midpoint width	10.9 ± 3.9	59.0 ± 15.5

*Demographic and morphologic variables of measured specimens are summarized below. The raw measurements are reported in mm. Surface area is reported in mm^2^. Normalized values were divided by clavicle length (L), except for CC facet along width, which was divided by clavicle width (W). These values are reported multiplied by 100*.

**Table 4:** Univariate models of CCJ morphology.

**Effect**	**Mean ± SD in Reference group**	**Mean ± SD in Comparative group**	**Effect size (CI)**	***p*-value**
Elevation				
10 year increase in age	–	–	0.1 (0.0, 0.2)	0.001
Male gender	1.1 ± 0.9	1.2 ± 0.9	0.1 (−0.1, 0.4)	0.219
AA race	1.2 ± 0.8	1.2 ± 1.0	0.1 (−0.1, 0.3)	0.537
Surface Area				
10 year increase in age	–	–	3.8 (2.0, 5.6)	<0.001
Male gender	49.5 ± 22.9	53.1 ± 28.0	3.1 (−4.0, 10.3)	0.386
AA race	52.4 ± 26.2	52.2 ± 27.6	−0.6 (−7.0, 5.9)	0.864
Midpoint Along Length				
10 year increase in age	–	–	−0.2 (−0.4, 0.0)	0.103
Male gender	25.5 ± 2.9	25.0 ± 2.9	−0.5 (−1.3, 0.4)	0.295
AA race	25.4 ± 2.5	24.9 ± 3.1	−0.5 (−1.3, 0.2)	0.125
Midpoint Along Width				
10 year increase in age	–	–	0.7 (−0.7, 2.1)	0.318
Male gender	54.4 ± 16.8	60.4 ± 14.8	5.9 (1.0, 10.8)	0.017
AA race	59.7 ± 16.3	58.5 ± 14.8	−0.8 (−4.8, 3.2)	0.695

*The normalized variable is the outcome and each covariate is modeled for it. For dichotomous variables, the mean ± SD is shown for each group (female and non-AA are the reference groups). For all variables, the effect of that variable on the outcome is shown with CI and p-value*.

**Figure 2:**
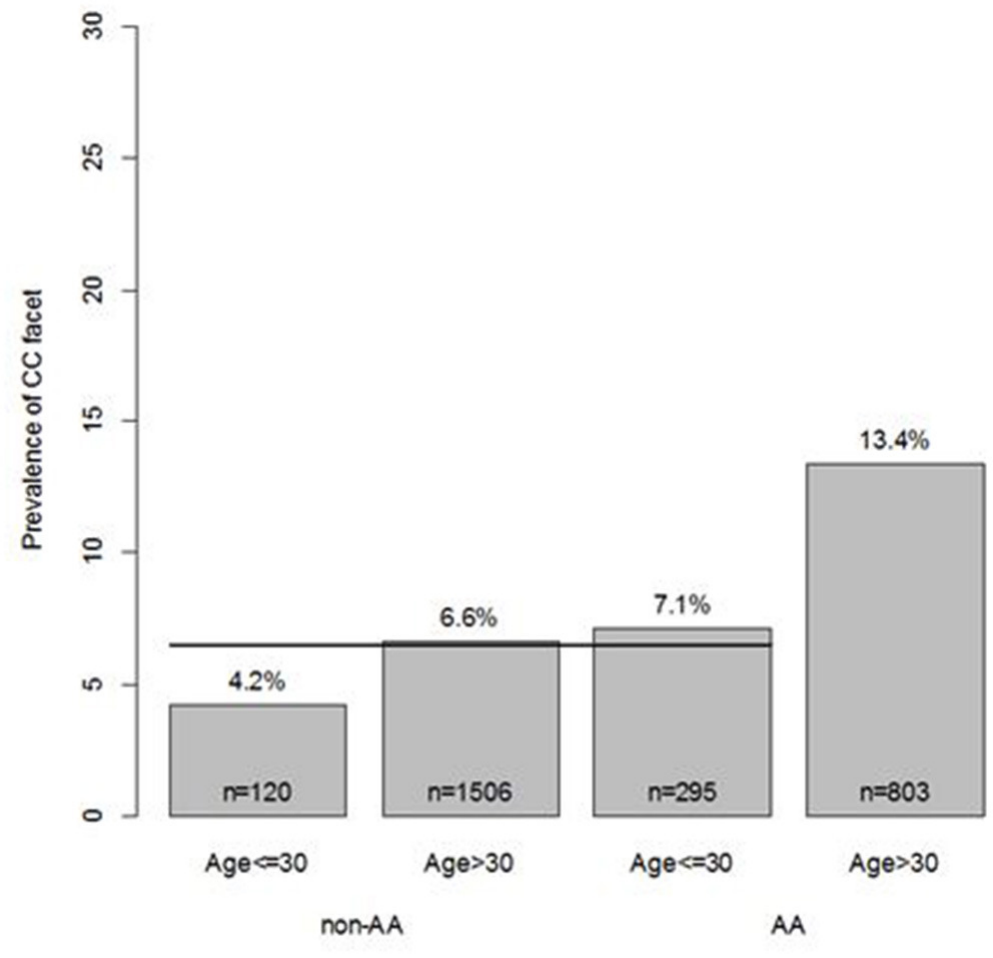
Multivariable modeling of CC facet. In multivariable modeling, considering all models with 2-way interactions, the model that identified the AA and age >30 group as different from the other groups was found to be the best predictor of CC facet. No other effects (other variables or other interactions) were found to improve upon this model.

In the multivariable model evaluation of age effects, the prevalence of CCJ in the group of African-American and greater than 30 years old (13.4%) was significantly different from the prevalence in all other groups (6.5%) [OR = 2.23 (1.70, 2.93), *p* < 0.001] (Figure 2). The results were similar in the bilateral CCJ analysis. The prevalence of bilateral CCJ in African-American and greater than 30 years old group was 7.8%, which was much higher than the prevalence in all other groups (3.1%) [OR = 2.69 (1.86, 3.87), *p* < 0.001]. In each multivariable model, there was not enough evidence to conclude that the prevalence was different among African-American younger than 30, Caucasians older than 30, or Caucasians younger than 30.

Interestingly, normalized elevation of the CC facet is on average approximately 1% of the clavicle length and, for every 10-year increase in age, elevation increased by about 0.1% on average (*p* = 0.001). Normalized surface area of the CC facet is on average approximately 50% of clavicle length, and increased about 4% for a 10-year increase in age (*p* < 0.001). No significant gender or race effects were found for size.

Lastly, as the articulation with the CC facet occurs over a bony area of the coracoid that has just finished curving anteriorly, the coracoid facet tended to be a flatter, shallow, less raised surface, making measurements less reliable and were excluded from detailed analysis.

## Discussion

The CCJ is an anomalous articulation between the coracoid process of the scapula and the inferior surface of the clavicle. It has been proposed as a potential source of recurrent anterior-superior shoulder pain (3, 19). Previous studies have reported a wide range of prevalence of this joint from 0.04 to 40.7% (3–7). Additionally, there have been contrasting findings of population variance of its prevalence between gender and races. There have been no prior studies that sought to specifically characterize CCJ morphology and this study provides the largest osteological investigation of the clavicular facet of the CCJ (CC facet).

As noted previously, a wide variation in the observed prevalence of the CCJ has been reported in the literature. In small osteological studies, this has ranged from 0.3 to 10% (2, 3, 5, 6, 28). The reported prevalence in radiological studies has been much higher, ranging from 0 to 40.7% (3). In a study of 60 white and 180 black South African osteological samples, the overall CCJ prevalence was 9.6% – similar to our observed prevalence of 8.6% (5). With respect to gender, only one study has noted a much larger frequency of the CCJ in males compared to females (11:1) (8). Whereas in our study, females were more likely than males to have CCJ according to the univariate model, a fact not previously reported in the literature. However, in multivariable modeling, this difference was not found because of the relatively large proportion of African-Americans in the female group and small proportion of African-Americans in the male group. Finally, previous studies have not found a difference in the frequency of CCJ between races. Our findings contrast this, as the prevalence of CCJ was significantly lower in Caucasians than African-Americans. In fact, from our data, it appears that the interaction of age and race is a more likely candidate for differences in CCJ prevalence than is gender.

**Figure 3:**
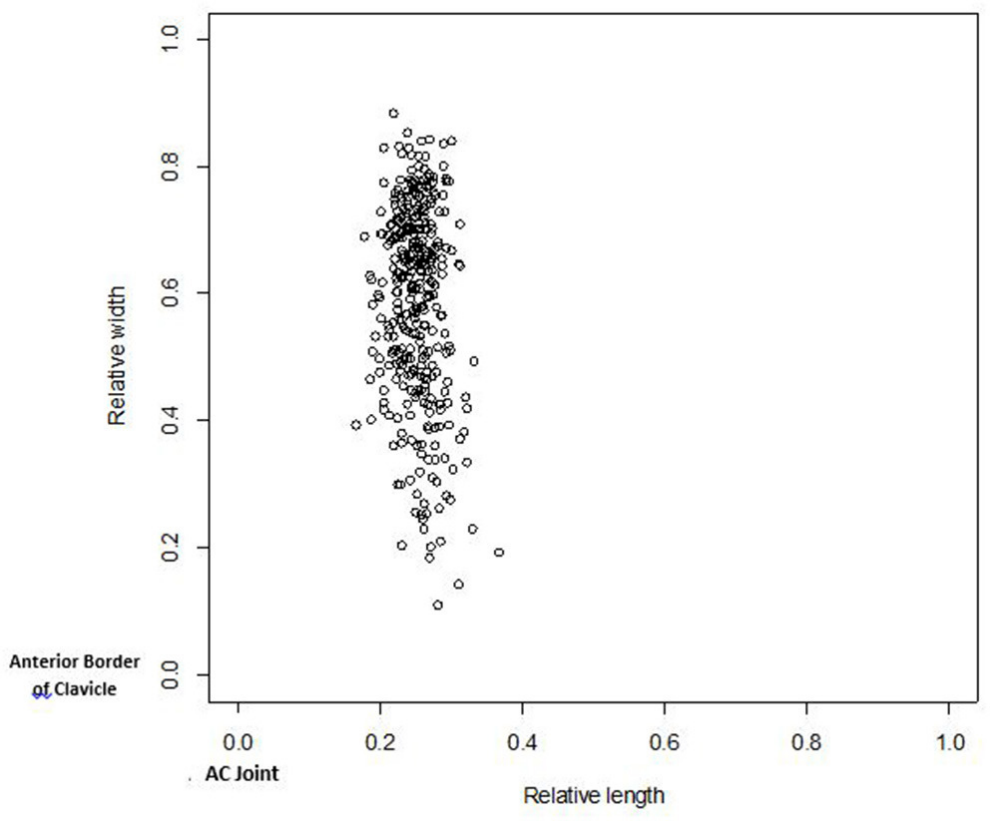
CC facet midpoint along width vs. midpoint along length. The midpoint of the facet along the width of the clavicle (relative to the width of the clavicle) is plotted vs. the midpoint of the facet along the length of the clavicle (relative to the length of the clavicle). The scale of 0–1 represents the entire length and width of the clavicle. For relative width, 0 is the anterior border and 1 is posterior border. For relative length, 0 is the point at the acromicoclavicular end and 1 is at the sternoclavicular end. Black circles are males, white circles are females.

Previous osteological studies have not characterized the location along the clavicle of this anomalous CC facet. The location of the CC facet along clavicle length was consistent across individuals in our study with an average location of 25% along the length of the clavicle from the AC joint. Importantly, the location of the facet along the width of the clavicle, or the anterior-posterior axis from the anterior border of the clavicle, varied substantially. Males had a significantly more posterior CC facet location than females did when normalized for clavicle width (Figure 3).

The etiology of the CCJ has been the subject of much debate, as one study suggested that CCJ occurrence is related to the aging process since the authors did not find one in individuals less than 40 (10). Kaur and Jit proposed that that the joint develops after the first decade of life (4). They studied the clavicles of both adults and children and did not find a CCJ in individuals younger than 13 years old. However, in a radiological study, a CCJ was observed in 2 children, ages 3 and 7 (6). Additionally, authors have noted that localized pressure and friction can induce metaplastic changes transforming connective tissue into cartilage (8). A recent study showed that anterolateral movement of the inferior angle of the scapula caused the conoid tubercle to collide with the trapezoid ligament on the superior surface of the coracoid process (29). Such contact could theoretically induce the development of a CCJ. Our findings are consistent with this hypothesis as both the elevation and surface area of the CC facet increased with age. Moreover, no individuals younger than 18 years old had a CCJ and its prevalence was greater in individuals older than 30 years (9.0%), as opposed to younger individuals (6.3%). This suggests that the CCJ is not a congenital abnormality, but instead develops at some point in life and continues to grow after its appearance.

Clinically, the presence of the CCJ has been suggested to predispose the sternoclavicular and acromioclavicular joints to degenerative changes (2, 12, 17, 24). But, this remains unclear, along with whether the CCJ itself can develop osteoarthritis (12, 17, 24). The effect of the CCJ on adjacent joint arthritis is beyond the scope of this paper, but knowledge of its morphology and prevalence patterns may be useful particularly in patients with recalcitrant anterior shoulder pain or an unclear diagnosis with radiographic evidence of a CCJ, because excision has been a successful surgical option for a painful CCJ (9, 12–27). Our study found that the location of the CC facet is relatively consistent with respect to clavicle length from the AC joint, but location varies along clavicle width, with males having a more posterior facet location. Building on the results of the current study, future studies may seek to elucidate the effect of a CCJ on shoulder joint biomechanics and the effect of its excision on resultant shoulder kinematics.

There are important limitations of this study to address. One was the lack of cartilage or soft tissue in our analysis as we only investigated bony changes. Interestingly, histological examinations of the CCJ have revealed variations in the extent of the CCJ. Pillay first classified CCJ into subtypes: grade 1 (fully developed joint with cartilage-lined articular surfaces and an entire articular capsule) and grade 2 (incomplete joint without cartilage or capsule, but articulation present in between the clavicle and coracoid process) (9). Interestingly, a grade 2 finding is still classified as a CCJ despite the absence of identifiable cartilage. Even though we were not able to assess the condition or presence of cartilage, these findings of different CCJ subtypes appear to agree with our observations. Some subjects had fully formed bony articulations with well-defined facets on both the clavicle and coracoid process, while others only had small clavicular facets. Moreover, our study did not investigate the presence and morphology of the coracoid component to the CCJ. Notably, its presence is not consistently defined or evaluated in other studies investigating the CCJ (2). We found that the coracoid facet tended to be a flatter, shallow, less raised surface, making measurements less reliable. Future sides will seek to clarify the prevalence of the coracoid facet in CCJ but may seek to do in a cadaveric study as the osseous changes are less apparent in an osteological model and better data is needed in describing the articulating soft tissue architecture. This will also help us better understand the true etiology of the CCJ. Secondly, there was no clinical information available to determine if individuals with a CCJ were symptomatic. As this was a strictly anatomical study, this information was unnecessary. Lastly, this population may not be completely generalizable and may have had different environmental, social, and nutritional factors than today's population. For example, males in this study tended to be slightly shorter in stature and females tended to be slightly taller than today's population (30). Additionally, this population had a very large percentage of males (82%) which may introduce a sampling bias. With those factors considered, we believe that with our large sample collection can still be reasonably extrapolated to be representative of today's population, more so than any other osteological study to date.

## Data Availability Statement

The raw data supporting the conclusions of this article will be made available by the authors, without undue reservation.

## Author Contributions

EH data analysis and manuscript writing. LS and CT data collection and manuscript writing. BD and JI critical editing of the manuscript. SM supervisor, oversaw data analysis, drafted, and critically revised manuscript. All authors contributed to the article and approved the submitted version.

## Conflict of Interest

The authors declare that the research was conducted in the absence of any commercial or financial relationships that could be construed as a potential conflict of interest.

## Publisher's Note

All claims expressed in this article are solely those of the authors and do not necessarily represent those of their affiliated organizations, or those of the publisher, the editors and the reviewers. Any product that may be evaluated in this article, or claim that may be made by its manufacturer, is not guaranteed or endorsed by the publisher.
